# Some isomorphic properties of *m*-polar fuzzy graphs with applications

**DOI:** 10.1186/s40064-016-3783-z

**Published:** 2016-12-20

**Authors:** Ganesh Ghorai, Madhumangal Pal

**Affiliations:** Department of Applied Mathematics with Oceanology and Computer Programming, Vidyasagar University, Midnapore, 721 102 India

**Keywords:** *m*-Polar fuzzy graphs, Order and size, Busy and free vertices, Isomorphisms, Self complement and weak self complement, 5-Polar fuzzy evaluation graph

## Abstract

The theory of graphs are very useful tool in solving the combinatorial problems in different areas of computer science and computational intelligence systems. In this paper, we present a frame work to handle *m*-polar fuzzy information by combining the theory of *m*-polar fuzzy sets with graphs. We introduce the notion of weak self complement *m*-polar fuzzy graphs and establish a necessary condition for *m*-polar fuzzy graph to be weak self complement. Some properties of self complement and weak self complement *m*-polar fuzzy graphs are discussed. The order, size, busy vertices and free vertices of an *m*-polar fuzzy graphs are also defined and proved that isomorphic *m*-polar fuzzy graphs have same order, size and degree. Also, we have presented some results of busy vertices in isomorphic and weak isomorphic *m*-polar fuzzy graphs. Finally, a relative study of complement and operations on *m*-polar fuzzy graphs have been made. Applications of *m*-polar fuzzy graph are also given at the end.

## Background

After the introduction of fuzzy sets by Zadeh (1965), fuzzy set theory have been included in many research fields. Since then, the theory of fuzzy sets has become a vigorous area of research in different disciplines including medical and life sciences, management sciences, social sciences engineering, statistic, graph theory, artificial intelligence, signal processing, multi agent systems, decision making and automata theory. In a fuzzy set, each element is associated with a membership value selected from the interval [0, 1]. Zhang (1994, 1998) introduced the concept of bipolar fuzzy sets. Instead of using particular membership value as in fuzzy sets, *m*-polar fuzzy set can be used to represent uncertainty of a set more perfectly. Chen et al. (2014) introduced the notion of *m*-polar fuzzy set as a generalization of fuzzy set theory. The membership value in *m*-polar fuzzy set is more expressive in capturing uncertainty of data.

An *m*-polar fuzzy set on a non-void set *X* is a mapping $\mu :X\rightarrow [0,1]^m$. The idea behind this is that “multipolar information” exists because data of real world problems are sometimes come from multiple agents. *m*-polar fuzzy sets allow more graphical representation of vague data, which facilitates significantly better analysis in data relationships, incompleteness, and similarity measures. Graph theory besides being a well developed branch of Mathematics, it is an important tool for mathematical modeling. Realizing the importance, Rosenfeld (1975) introduced the concept of fuzzy graphs, Mordeson and Nair (2000) discussed about the properties of fuzzy graphs and hypergraphs. After that, the operation of union, join, Cartesian product and composition on two fuzzy graphs was defined by Mordeson and Peng (1994). Sunitha and Vijayakumar (2002) further studied the other properties of fuzzy graphs. The concept of weak isomorphism, co-weak isomorphism and isomorphism between fuzzy graphs was introduced by Bhutani (1989). Later many researchers have worked on fuzzy graphs like in Bhutani et al. (2004); Al-Hawary (2011); Koczy (1992); Lee-kwang and Lee (1995); Nagoorgani and Radha (2008), Samanta and Pal (2011a, b, 2013, 2014, 2015). Akram (2011, 2013) introduced and defined different operations on bipolar fuzzy graphs. Again, Rashmanlou et al. (2015a, 2015b, 2016) studied bipolar fuzzy graphs with categorical properties, product of bipolar fuzzy graphs and their degrees, etc. Using these concepts many research is going on till date on bipolar fuzzy graphs such as Ghorai and Pal (2015b), Samanta and Pal (2012a, b, 2014), Yang et al. (2013). Chen et al. (2014) first introduced the concept of *m*-polar fuzzy graphs. Then Ghorai and Pal (2016a) presented properties of generalized *m*-polar fuzzy graphs, defined many operations and density of *m*-polar fuzzy graphs (2015a), introduced the concept of *m*-polar fuzzy planar graphs (2016b) and defined faces and dual of *m*-polar fuzzy planar graphs (2016c). Akram and Younas (2015), Akram et al. (2016) introduced irregular *m*-polar fuzzy graphs and metrics in *m*-polar fuzzy graphs. In this paper, weak self complement *m*-polar fuzzy graphs is defined and a necessary condition is mentioned for an *m*-polar fuzzy graph to be weak self complement. Some properties of self complement and weak self complement *m*-polar fuzzy graphs are discussed. The order, size, busy vertices and free vertices of an *m*-polar fuzzy graphs are also defined and proved that isomorphic *m*-polar fuzzy graphs have same order, size and degree. Also, we have proved some results of busy vertices in isomorphic and weak isomorphic *m*-polar fuzzy graphs. Finally, a relative study of complement and operations on *m*-polar fuzzy graphs have been made.

## Preliminaries

First of all we give the definitions of *m*-polar fuzzy sets, *m*-polar fuzzy graphs and other related definitions from the references (Al-Harary 1972; Lee 2000).

Throughout the paper, $[0,1]^m$ (*m*-power of [0, 1]) is considered to be a poset with point-wise order $\le$, where *m* is a natural number. $\le$ is defined by $x\le y \Leftrightarrow$ for each $i=1,2,\ldots ,m$, $p_i(x)\le p_i(y)$ where $x, y\in [0,1]^m$ and $p_i:[0,1]^m \rightarrow [0,1]$ is the *i*th projection mapping.

As a generalization of bipolar fuzzy sets, Chen et al. (2014) defined the *m*-polar fuzzy sets in 2014.

### **Definition 1**

(Chen et al. 2014) Let *X* be a non-void set. An *m*-polar fuzzy set on *X* is defined as a mapping $\mu :X\rightarrow [0,1]^m$.

The *m*-polar fuzzy relation is defined below.

### **Definition 2**

(Ghorai and Pal 2016a) Let *A* be an *m*-polar fuzzy set on a set *X*. An *m*-polar fuzzy relation on *A* is an *m*-polar fuzzy set *B* of $X\times X$ such that $p_i\circ B(x,y)\le min\{p_i\circ A(x),p_i\circ A(y)\}$ for all $x,y\in X$, $i=1,2,\ldots ,m$. *B* is called symmetric if $B(x,y)=B(y,x)$ for all $x,y\in X$.

We define an equivalence relation $\sim$ on $V\times V-\{(x,x): x\in V\}$ as follows:

We say $(x_1,y_1)\sim (x_2,y_2)$ if and only if either $(x_1,y_1)=(x_2,y_2)$ or $x_1=y_2$ and $y_1=x_2$. Then we obtain an quotient set denoted by $\widetilde{V^2}$. The equivalence class containing the element (*x*, *y*) will be denoted as *xy* or *yx*.

We assume that $G^*=(V,E)$ is a crisp graph and $G=(V,A,B)$ is an *m*-polar fuzzy graph of $G^*$ throughout this paper.

Chen et al. (2014) first introduced *m*-polar fuzzy graph. We have modified their definition and introduce generalized *m*-polar fuzzy graph as follows.

### **Definition 3**

(Chen et al. 2014; Ghorai and Pal 2016a) An *m*-polar fuzzy graph (or generalized *m*-polar fuzzy graph) of $G^*=(V,E)$ is a pair $G=(V,A,B)$ where $A: V\rightarrow [0,1]^m$ is an *m*-polar fuzzy set in *V* and $B: \widetilde{V^2}\rightarrow [0,1]^m$ is an *m*-polar fuzzy set in $\widetilde{V^2}$ such that $p_i\circ B(xy)\le min\{p_i\circ A(x),p_i\circ A(y)\}$ for all $xy\in \widetilde{V^2}$, $i=1,2,\ldots ,m$ and $B(xy)={\mathbf{0}}$ for all $xy\in \widetilde{V^2}-E$, $\big ({\mathbf{0}} =(0,0,\ldots ,0)$ is the smallest element in $[0,1]^m\big )$. We call *A* as the *m*-polar fuzzy vertex set of *G* and *B* as the *m*-polar fuzzy edge set of *G*.

### *Example 4*

Let $G^*=(V, E)$ be a crisp graph where $V=\{u_1, u_2, u_3, u_4\}$ and $E=\{u_1u_2, u_2u_3, u_3u_4, u_4u_1\}$. Then, $G=(V, A, B)$ be a 3-polar fuzzy graph of $G^*$ where $A=\left\{ \frac{\langle 0.5, 0.7, 0.8\rangle }{u_1}, \frac{\langle 0.4, 0.7, 0.8\rangle }{u_2}, \frac{\langle0.7, 0.6, 0.8\rangle}{u_3}, \frac{\langle0.3, 0.6, 0.9\rangle}{u_4}\right\}$ and $B=\left\{ \frac{\langle 0.4, 0.6, 0.7\rangle }{u_1u_2}, \frac{\langle 0.3, 0.6, 0.5\rangle }{u_2u_3}, \frac{\langle 0.2, 0.5, 0.6\rangle }{u_3u_4}, \frac{\langle 0.2, 0.4, 0.8\rangle }{u_4u_1}, \frac{\langle 0, 0, 0\rangle }{u_1u_3}, \frac{\langle 0, 0, 0\rangle }{u_4u_2}\right\}$.

Ghorai and Pal (2016a) introduced many operations on *m*-polar fuzzy graphs such as Cartesian product, composition, union and join which are given below.

### **Definition 5**

(Ghorai and Pal 2016a) The Cartesian product of two *m*-polar fuzzy graphs $G_1=(V_1,A_1,B_1)$ and $G_2=(V_2,A_2,B_2)$ of the graphs $G^*_1$ and $G^*_2$ respectively is denoted as a pair $G_1\times G_2=(V_1\times V_2,A_1\times A_2,B_1\times B_2)$ such that for $i=1,2,\ldots ,m$

(i) $$p_i\circ (A_1\times A_2)(x_1,x_2)= min\{p_i\circ A_1(x_1),p_i\circ A_2(x_2)\}$$ for all $$(x_1,x_2)\in V_1\times V_2$$.
(ii) $p_i\circ (B_1\times B_2)((x,x_2)(x,y_2))= min\{p_i\circ A_1(x),p_i\circ B_2(x_2y_2)\}$ for all $x\in V_1$, $x_2y_2\in E_2$.
(iii) $p_i\circ (B_1\times B_2)((x_1,z)(y_1,z))= min\{p_i\circ B_1(x_1y_1),p_i\circ A_2(z)\}$ for all $z\in V_2$, $x_1y_1\in E_1$.
(iv) $p_i\circ (B_1\times B_2)((x_1,x_2)(y_1,y_2))=0$ for all $(x_1,x_2)(y_1,y_2)\in \widetilde{(V_1\times V_2)^2}-E$.

### **Definition 6**

(Ghorai and Pal 2016a) The composition of two *m*-polar fuzzy graphs $G_1=(V_1,A_1,B_1)$ and $G_2=(V_2,A_2,B_2)$ of the graphs $G^*_1=(V_1,E_1)$ and $G^*_2=(V_2,E_2)$ respectively is denoted as a pair $G_1[G_2]=(V_1\times V_2,A_1\circ A_2,B_1\circ B_2)$ such that for $i=1,2,\ldots ,m$

(i) $$p_i\circ (A_1\circ A_2)(x_1,x_2)= min\{p_i\circ A_1(x_1),p_i\circ A_2(x_2)\}$$ for all $$(x_1,x_2)\in V_1\times V_2$$.
(ii) $p_i\circ (B_1\circ B_2)((x,x_2)(x,y_2))= min\{p_i\circ A_1(x),p_i\circ B_2(x_2y_2)\}$ for all $x\in V_1$, $x_2y_2\in E_2$.
(iii) $p_i\circ (B_1\circ B_2)((x_1,z)(y_1,z))= min\{p_i\circ B_1(x_1y_1),p_i\circ A_2(z)\}$ for all $z\in V_2$, $x_1y_1\in E_1$.
(iv) $p_i\circ (B_1\circ B_2)((x_1,x_2)(y_1,y_2)) =min\{p_i\circ A_2(x_2),p_i\circ A_2(y_2),p_i\circ B_1(x_1y_1)\}$ for all $(x_1,x_2)(y_1,y_2)\in E^0-E$.
(v) $$p_i\circ (B_1\circ B_2)((x_1,x_2)(y_1,y_2))=0$$ for all $$(x_1,x_2)(y_1,y_2)\in \widetilde{(V_1\times V_2)^2}-E^0$$.

### **Definition 7**

(Ghorai and Pal 2016a) The union $G_1\cup G_2=(V_1\cup V_2,A_1\cup A_2,B_1\cup B_2)$ of the *m*-polar fuzzy graphs $G_1=(V_1,A_1,B_1)$ and $G_2=(V_2,A_2,B_2)$ of $G^*_1$ and $G^*_2$ respectively is defined as follows: for $i=1,2,\ldots ,m$

(i) $$p_i\circ (A_1\cup A_2)(x)=\left\{ \begin{array}{ll} p_i\circ A_1(x) &{}\quad {\text {if}}\; x\in V_1-V_2\\ p_i\circ A_2(x) &{}\quad {\text {if}}\; x\in V_2-V_1\\ max\{p_i\circ A_1(x),p_i\circ A_2(x)\} &{}\quad {\text {if}}\; x\in V_1\cap V_2. \end{array}\right. $$
(ii) $$p_i\circ (B_1\cup B_2)(xy)=\left\{ \begin{array}{ll} p_i\circ B_1(xy) &{}\quad {\text {if}}\; xy\in E_1-E_2\\ p_i\circ B_2(xy) &{}\quad {\text {if}}\; xy\in E_2-E_1\\ max\{p_i\circ B_1(xy),p_i\circ B_2(xy)\} &{}\quad {\text {if}}\; xy\in E_1\cap E_2. \end{array}\right. $$
(iii) $$p_i\circ (B_1\cup B_2)(xy)=0$$ if $$xy\in \widetilde{(V_1\times V_2)^2}-E_1\cup E_2$$.

### **Definition 8**

(Ghorai and Pal 2016a) The join of the *m*-polar fuzzy graphs $G_1= (V_1,A_1,B_1)$ and $G_2= (V_2,A_2,B_2)$ of $G^*_1$ and $G^*_2$ respectively is defined as a pair $G_1+ G_2= (V_1\cup V_2,A_1+ A_2,B_1+ B_2)$ such that for $i=1,2,\ldots ,m$

(i) $$p_i\circ (A_1+A_2)(x)=p_i\circ (A_1\cup A_2)(x)$$ if $$x\in V_1\cup V_2$$.
(ii) $$p_i\circ (B_1+B_2)(xy)=p_i\circ (B_1\cup B_2)(xy)$$ if $$xy\in E_1\cup E_2$$.
(iii) $p_i\circ (B_1+B_2)(xy)=min\{p_i\circ A_1(x),p_i\circ A_2(y)\}$ if $xy\in E^\prime$, where $E^\prime$ denotes the set of all edges joining the vertices of $V_1$ and $V_2$.
(iv) $$p_i\circ (B_1+B_2)(xy)=0$$ if $$xy\in \widetilde{(V_1\times V_2)^2}-E_1\cup E_2\cup E^\prime $$.

### *Remark 9*

Later on, Akram et al. (2016) applied the concept of *m*-polar fuzzy sets on graph structure and also defined the above operations on them.

Different types of morphism are defined on *m*-polar fuzzy graphs by Ghorai and Pal (2016a).

### **Definition 10**

(Ghorai and Pal 2016a) Let $G_1=(V_1,A_1,B_1)$ and $G_2=(V_2,A_2,B_2)$ be two *m*-polar fuzzy graphs of the graphs $G^*_1= (V_1,E_1)$ and $G^*_2= (V_2,E_2)$ respectively. A homomorphism between $G_1$ and $G_2$ is a mapping $\phi :V_1\rightarrow V_2$ such that for each $i=1,2,\ldots ,m$

(i) $$p_i\circ A_1(x_1)\le p_i\circ A_2(\phi (x_1))$$ for all $$x_1\in V_1$$,
(ii) $p_i\circ B_1(x_1y_1)\le p_i\circ B_2(\phi (x_1)\phi (y_1))$ for all $x_1y_1\in \widetilde{V^2_1}$.

$\phi$ is said to be an isomorphism if it is a bijective mapping and for $i=1,2,\ldots ,m$

(i) $$p_i\circ A_1(x_1)= p_i\circ A_2(\phi (x_1))$$ for all $$x_1\in V_1$$,
(ii) $p_i\circ B_1(x_1y_1)= p_i\circ B_2(\phi (x_1)\phi (y_1))$ for all $x_1y_1\in \widetilde{V^2_1}$.

In this case, we write $G_1\cong G_2$.

### **Definition 11**

(Ghorai and Pal 2016a) A weak isomorphism between $G_1=(V_1,A_1,B_1)$ and $G_2=(V_2,A_2,B_2)$ is a bijective mapping $\phi :V_1\rightarrow V_2$ such that

(i) $$\phi $$ is a homomorphism,
(ii) $p_i\circ A_1(x_1)= p_i\circ A_2(\phi (x_1))$ for all $x_1\in V_1$, for each $i=1,2,\ldots ,m$.

### **Definition 12**

(Ghorai and Pal 2016a) $G=(V,A,B)$ is called strong if $p_i\circ B(xy)=min\{p_i\circ A(x),p_i\circ A(y)\}$ for all $xy\in E$, $i=1,2,\ldots ,m$.

A strong *m*-polar fuzzy graph *G* is called self complementary if $G\cong \overline{G}$.

Degree of a vertex in an *m*-polar fuzzy graph is defined as below.

### **Definition 13**

(Akram and Younas 2015) The neighborhood degree of a vertex *v* in the *m*-polar fuzzy graph *G* is denoted as $deg(v)=\big (p_1\circ deg(v), p_2\circ deg(v), \ldots , p_m\circ deg(v)\big )$ where $$p_i\circ deg(v)=\sum \nolimits _{\begin{array}{c} u\ne v\\ uv\in E \end{array}}p_i\circ {B}(uv)$$, $i=1,2,\ldots ,m$.

### *Remark 14*

If $G_1=(V_1, A_1, B_1)$ and $G_2=(V_2, A_2, B_2)$ are two *m*-polar fuzzy graphs. Then the canonical projection maps $\pi _1: V_1\times V_2\rightarrow V_1$ and $\pi _2: V_1\times V_2\rightarrow V_2$ are indeed homomorphisms from $G_1\times G_2$ to $G_1$ and $G_1\times G_2$ to $G_2$ respectively. This can be seen as follows:

$p_i\circ (A_1\times A_2)(x_1, x_2)=min\{p_i\circ A_1(x_1), p_i\circ A_2(x_2)\}\le p_i\circ A_1(x_1)=p_i\circ A_1(\pi _1(x_1, x_2))$ for all $(x_1, x_2)\in V_1\times V_2$ and $p_i\circ (B_1\times B_2)((x_1, z)(y_1, z))=min\{p_i\circ B_1(x_1y_1), p_i\circ A_2(z)\}\le p_i\circ B_1(x_1y_1)=p_i\circ B_1(\pi _1(x_1, z)\pi _1(y_1, z))$ for all $z\in V_2$ and $x_1y_1\in E_1$. In a similar way we can check the other conditions also. This shows that the canonical projection maps $\pi _1: V_1\times V_2\rightarrow V_1$ is a homomorphism from $G_1\times G_2$ to $G_1$.

## Weak self complement *m*-polar fuzzy graphs

Self complement *m*-polar fuzzy graphs have many important significant in the theory of *m*-polar fuzzy graphs. If an *m*-polar fuzzy graph is not self complement then also we can say that it is self complement in some weaker sense. Simultaneously we can establish some results with this graph. This motivates to define weak self complement *m*-polar fuzzy graphs.

### **Definition 15**

Let $G=(V,A,B)$ be an *m*-polar fuzzy graph of the crisp graph $G^*=(V,E)$. The complement of *G* is an *m*-polar fuzzy graph $\overline{G}=(V,\overline{A},\overline{B})$ of $\overline{G^*}=(V,\widetilde{V^2})$ such that $\overline{A}=A$ and $\overline{B}$ is defined by $p_i\circ \overline{B}(xy)=min\{p_i\circ A(x), p_i\circ A(y)\}- p_i\circ B(xy)$ for $xy\in \widetilde{V^2}$, $i=1,2,\ldots ,m$.

### *Example 16*

**Fig. 1:**
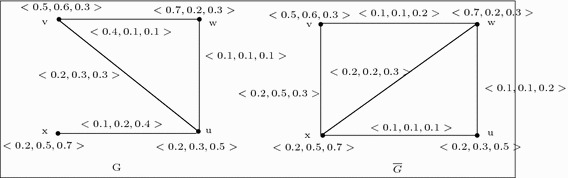
*G* and it’s complement $\overline{G}$

Let $G=(V,A,B)$ be a 3-polar fuzzy graph of the graph $G^*=(V,E)$ where $V=\{u,v,w,x\}$, $E=\{uv,vw,wu,ux\}$, $A=\left\{ \frac{\langle 0.2,0.3,0.5\rangle }{u},\frac{\langle 0.5,0.6,0.3\rangle }{v},\frac{\langle 0.7,0.2,0.3\rangle }{w},\,\frac{\langle 0.2,0.5,0.7\rangle }{x} \right\}$, $B=\left\{ \frac{\langle 0.2,0.3,0.3\rangle }{uv},\frac{\langle 0.4,0.1,0.1\rangle }{vw},\frac{\langle 0.1,0.1,0.1\rangle }{wu}, \frac{\langle 0.1,0.2,0.4\rangle }{xu},\frac{\langle 0,0,0\rangle }{xv},\frac{\langle 0,0,0\rangle }{wx}\right\}$. Then by Definition 15, we have constructed the complement $\overline{G}$ of *G* which is shown in Fig. 1.

### *Remark 17*

Let $\overline{\overline{G}}=(V,\overline{\overline{A}},\overline{\overline{B}})$ be the complement of $\overline{G}$ where $\overline{\overline{A}}=\overline{A}=A$ and

$$\begin{aligned} p_i\circ \overline{\overline{B}}(uv)&=  {} min\{p_i\circ \overline{A}(u),p_i\circ \overline{A}(v)\}-p_i\circ \overline{B}(uv)\\&=  {} min\{p_i\circ A(u),p_i\circ A(v)\}-\{min\{p_i\circ A(u), p_i\circ A(v)\}- p_i\circ B(uv)\}\\&=  {} p_i\circ B(uv)\quad {\hbox {for}}\; uv\in \widetilde{V^2},\, i=1,2,\ldots ,m. \end{aligned}$$

Hence, $$\overline{\overline{G}}=G$$.

### **Definition 18**

The *m*-polar fuzzy graph $G=(V,A,B)$ is said to be weak self complement if there is a weak isomorphism from *G* onto $\overline{G}$. In other words, there exist a bijective homomorphism $\phi : G \rightarrow \overline{G}$ such that for $i=1,2,\ldots ,m$

(i) $$p_i\circ A(u)= p_i\circ \overline{A}(\phi (u))$$ for all $$u\in V$$,
(ii) $p_i\circ B(uv)\le p_i\circ \overline{B}(\phi (u)\phi (v))$ for all $uv\in \widetilde{V^2}$.

### *Example 19*

**Fig. 2:**
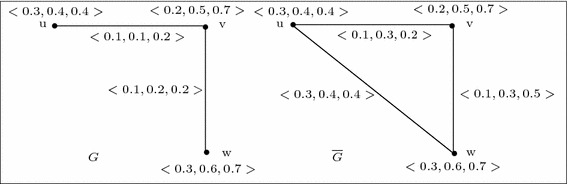
Weak self complement 3-polar fuzzy graphs

Let $G=(V,A,B)$ be a 3-polar fuzzy graph of the graph ${G^*}=(V,E)$ where $V=\{u,v,w\}$, $E=\{uv,vw\}$, $A=\left\{ \frac{\langle 0.3,0.4,0.4\rangle }{u},\frac{\langle 0.2,0.5,0.7\rangle }{v},\frac{\langle 0.3,0.6,0.7\rangle }{w}\right\}$, $B=\left\{ \frac{\langle 0.1,0.1,0.2\rangle }{uv},\frac{\langle 0.1,0.2,0.2\rangle }{vw},\frac{\langle 0,0,0\rangle }{wu}\right\}$. Then $\overline{G}=(V,\overline{A},\overline{B})$ is also a 3-polar fuzzy graph where $\overline{A}=A$ and $\overline{B}=\left\{ \frac{\langle 0.1,0.3,0.2\rangle }{uv},\frac{\langle 0.1,0.3,0.5\rangle }{vw},\frac{\langle 0.3,0.4,0.4\rangle }{wu}\right\}$. We can easily verify that, the identity map is an weak isomorphism from *G* onto $\overline{G}$(see Fig. 2). Hence *G* is weak self complement.

In Ghorai and Pal (2015a), Ghorai and Pal proved that if *G* is a self complementary strong *m*-polar fuzzy graph then for all $xy\in \widetilde{V^2}$ and $i=1,2,\ldots ,m$

$$\begin{aligned} \sum _{x\ne y} p_i\circ B(xy)=\frac{1}{2} \sum _{x\ne y} min\{p_i\circ A(x),p_i\circ A(y)\}. \end{aligned}$$

The converse of the above result does not hold always.

### *Example 20*

For example, let us consider a 3-polar fuzzy graph $G=(V,A,B)$ of ${G^*}=(V,E)$ where $V=\{u,v,w\}$, $E=\{uv,vw,wu\}$, $A=\left\{ \frac{\langle 0.2,0.3,0.4\rangle }{u},\frac{\langle 0.4,0.5,0.6\rangle }{v},\frac{\langle 0.5,0.7,0.8\rangle }{w}\right\}$, $B=\left\{ \frac{\langle 0.2,0.3,0.4\rangle }{uv},\frac{\langle 0.1,0.2,0.2\rangle }{vw},\frac{\langle 0.1,0.05,0.1\rangle }{wu}\right\}$. Then we have the following

$$\begin{aligned}&p_1\circ B(uv)+p_1\circ B(vw)+p_1\circ B(wu)=0.2+0.1+0.1=0.4\; {\text {and}}\\&\frac{1}{2}\left[ min\{p_1\circ A(u),p_1\circ A(v)\}+min\{p_1\circ A(v),p_1\circ A(w)\}+min\{p_1\circ A(w),p_1\circ A(u)\}\right] \\&\quad = \frac{1}{2}[min\{0.2,0.4\}+min\{0.4,0.5\}+min\{0.5,0.2\}]=\frac{1}{2}(0.2+0.4+0.2)=0.4. \end{aligned}$$

So,

$$\begin{aligned} \sum _{u\ne v} p_1\circ B(uv)=0.4=\frac{1}{2} \sum _{u\ne v} min\{p_1\circ A(u),p_i\circ A(v)\}. \end{aligned}$$

Similarly,

$$\begin{aligned} \sum _{u\ne v} p_2\circ B(uv)=0.55=\frac{1}{2} \sum _{u\ne v} min\{p_2\circ A(u),p_2\circ A(v)\} \end{aligned}$$

and

$$\begin{aligned} \sum _{u\ne v} p_3\circ B(uv)=0.7=\frac{1}{2} \sum _{u\ne v} min\{p_3\circ A(u),p_3\circ A(v)\}. \end{aligned}$$

Hence for $i=1,2,3$ we have,

$$\begin{aligned} \sum _{u\ne v} p_i\circ B(uv)=\frac{1}{2} \sum _{u\ne v} min\{p_i\circ A(u),p_i\circ A(v)\}. \end{aligned}$$

**Fig. 3:**
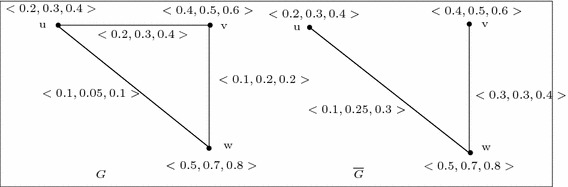
Example of 3-polar fuzzy graph *G* which is not self complement

But *G* is not self complementary as there exists no isomorphism from *G* onto $\overline{G}$ (see Fig. 3).

**Fig. 4:**
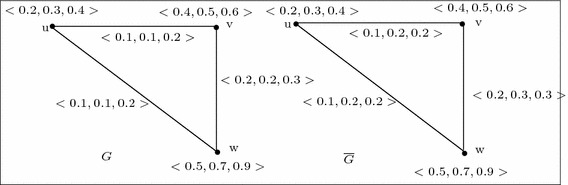
Example of 3-polar fuzzy graph *G* which is weak self complement

**Fig. 5:**
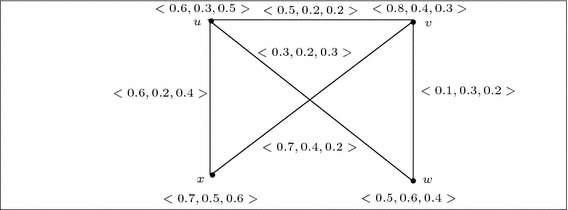
3-Polar fuzzy graph *G* and busy value of its vertices

**Fig. 6:**
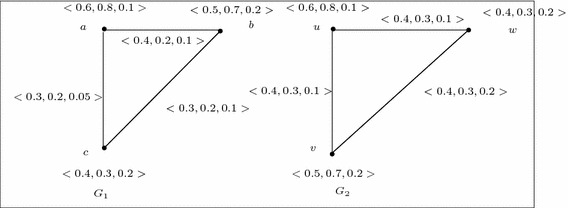
Weak isomorphic 3-polar fuzzy graphs $G_1$ and $G_2$

**Fig. 7:**
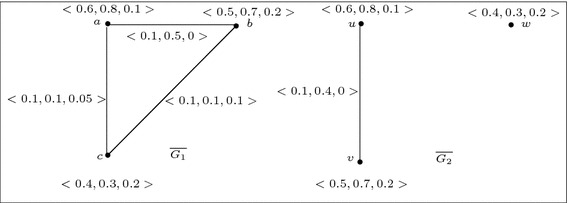
Example of weak isomorphic graphs whose complement is not weak isomorphic

**Fig. 8:**
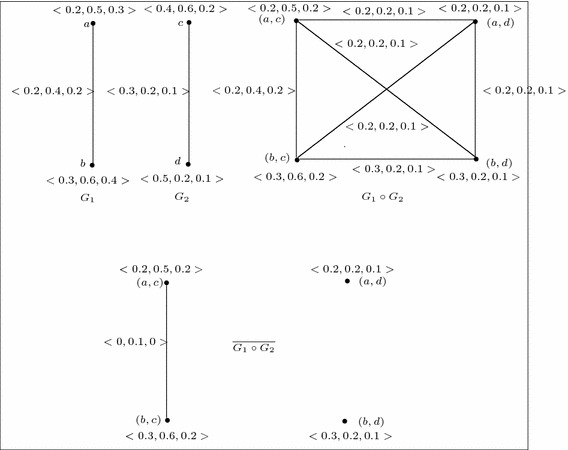
$$G_1$$, $$G_2$$, $$G_1\circ G_2$$ and $$\overline{G_1\circ G_2}$$

**Fig. 9:**
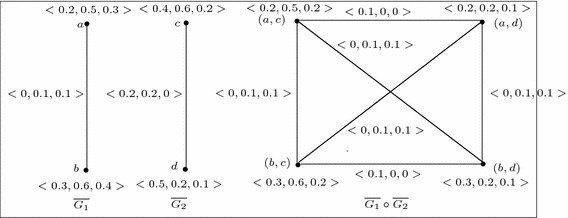
Example of 3-polar fuzzy graphs $G_1$ and $G_2$ where $\overline{G_1\circ G_2}\ncong \overline{G_1}\circ \overline{G_2}$

**Fig. 10:**
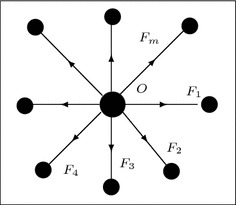
Graphical representation of tug of war

**Fig. 11:**
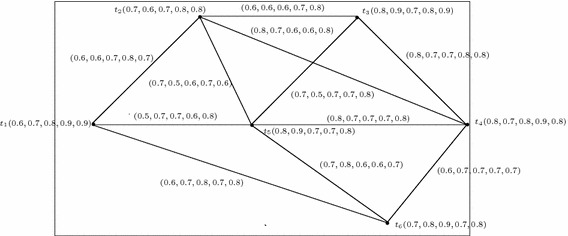
5-Polar fuzzy evaluation graph corresponding to the teacher’s evaluation by students

Now suppose an *m*-polar fuzzy graph $G=(V,A,B)$ is a weak self complement. Then the following inequality holds.

### **Theorem 21**

*Let* $G=(V,A,B)$ *be a weak self complement* *m*-*polar fuzzy graph of* ${G^*}$. *Then for* $i=1,2,\ldots ,m$

$$\begin{aligned} \sum _{x\ne y} p_i\circ B(xy)\le \frac{1}{2} \sum _{x\ne y} min\{p_i\circ A(x),p_i\circ A(y)\}. \end{aligned}$$

### *Proof*

Since *G* is weak self complement, therefore there exists a weak isomorphism $\phi : V \rightarrow V$ such that $p_i\circ A(x)= p_i\circ \overline{A}(\phi (x))$ for all $x\in V$ and $p_i\circ B(xy)\le p_i\circ \overline{B}(\phi (x)\phi (y))$ for all $xy\in \widetilde{V^2}$, $i=1,2,\ldots ,m$.

Using the above we have,

$$\begin{aligned}&p_i\circ B(xy)\le p_i\circ \overline{B}(\phi (x)\phi (y))=min\{p_i\circ A(x),p_i\circ A(y)\}- p_i\circ B(\phi (x)\phi (y))\\&{\hbox {i.e., }}p_i\circ B(xy)+p_i\circ B(\phi (x)\phi (y))\le min\{p_i\circ A(\phi (x)),p_i\circ A(\phi (y))\}. \end{aligned}$$

Therefore, for all $xy\in \widetilde{V^2}$, $i=1,2,\ldots ,m$

$$\begin{aligned}&\sum _{x\ne y} p_i\circ B(xy)+\sum _{x\ne y} p_i\circ B(\phi (x)\phi (y) \\&\quad \le \sum _{x\ne y} min\{p_i\circ A(\phi (x)),p_i\circ A(\phi (y))\} \\&\quad =\sum _{x\ne y} min\{p_i\circ A(x),p_i\circ A(y)\} \end{aligned}$$

i.e.,

$$\begin{aligned} 2\sum _{x\ne y} p_i\circ B(xy)\le \sum _{x\ne y} min\{p_i\circ A(x),p_i\circ A(y)\} \end{aligned}$$

i.e.,

$$\begin{aligned} \sum _{x\ne y} p_i\circ B(xy)\le \frac{1}{2}\sum _{x\ne y} min\{p_i\circ A(x),p_i\circ A(y)\}. \end{aligned}$$

$$\square $$

### *Remark 22*

The converse of the above theorem is not true in general. For example, consider the 3-polar fuzzy graph of Fig. 3. We see that for the 3-polar fuzzy graph *G*, the condition of Theorem 21 is satisfied. But, *G* is not weak self complementary as there is no weak isomorphism from *G* onto $\overline{G}$.

### **Theorem 23**

*If* $p_i\circ B(xy)\le \frac{1}{2} min\{p_i\circ A(x),p_i\circ A(y)\}$ *for all* $xy\in \widetilde{V^2}$, $i=1,2,\ldots ,m$ *then* *G* *is a weak self complement* *m*-*polar fuzzy graph*.

### *Proof*

Let $\overline{G}=(V,\overline{A},\overline{B})$ be the complement of *G* where $\overline{A}(x)=A(x)$ for all $x\in V$ and $p_i\circ \overline{B}(xy)=min\{p_i\circ A(x), p_i\circ A(y)\}- p_i\circ B(xy)$ for $xy\in \widetilde{V^2}$, $i=1,2,\ldots ,m$.

Let us now consider the identity map $I: V\rightarrow V$. Then $A(x)=A(I(x))=\overline{A}(I(x))$ for all $x\in V$ and

$$\begin{aligned} p_i\circ \overline{B}(I(x)I(y))&=  {} p_i\circ \overline{B}(xy)\\&=  {} min\{p_i\circ A(x), p_i\circ A(y)\}- p_i\circ B(xy)\\&\ge  {} min\{p_i\circ A(x), p_i\circ A(y)\}-\frac{1}{2} min\{p_i\circ A(x),p_i\circ A(y)\}\\&=  {} \frac{1}{2} min\{p_i\circ A(x),p_i\circ A(y)\}\ge p_i\circ B(xy). \end{aligned}$$

So, $p_i\circ B(xy)\le p_i\circ \overline{B}(I(x)I(y))$ for $i=1,2,\ldots ,m$ and $xy\in \widetilde{V^2}$. Hence, $I: V\rightarrow V$ is a weak isomorphism. $\square$

### *Example 24*

Consider the 3-polar fuzzy graph $G=(V,A,B)$ of ${G^*}=(V,E)$ where $V=\{u,v,w\}$, $E=\{uv,vw,wu\}$, $A=\left\{ \frac{\langle 0.2,0.3,0.4\rangle }{u},\frac{\langle 0.4,0.5,0.6\rangle }{v},\frac{\langle 0.5,0.7,0.9\rangle }{w}\right\}$, $B=\left\{ \frac{\langle 0.1,0.1,0.2\rangle }{uv},\frac{\langle 0.2,0.2,0.3\rangle }{vw},\frac{\langle 0.1,0.1,0.2\rangle }{wu}\right\}$. We see that for each $i=1,2,3$ and $xy\in \widetilde{V^2}$, $p_i\circ B(xy)\le \frac{1}{2} min\{p_i\circ A(x),p_i\circ A(y)\}$ .

Also, consider the complement of *G* of Fig. 4. Let us now consider the identity mapping $I: G\rightarrow \overline{G}$ such that $I(u)=u$ for all $u\in V$. Then, *I* is the required weak isomorphism from *G* onto $\overline{G}$. Hence, *G* is weak self complementary.

## Order, size and busy value of vertices of *m*-polar fuzzy graphs

In this section, the order, size, busy value of vertices of an *m*-polar fuzzy graph is defined.

### **Definition 25**

The order of the *m*-polar fuzzy graph $G=(V,A,B)$ is denoted by |*V*| (or *O*(*G*)) where

$$\begin{aligned} O(G)=|V|=\sum _{x\in V} \frac{1+ \sum \nolimits _{i=1}^{m} p_i\circ A(x)}{2}. \end{aligned}$$

The size of *G* is denoted by |*E*| (or *S*(*G*)) where

$$\begin{aligned} S(G)=|E|=\sum _{xy\in E} \frac{1+ \sum \nolimits _{i=1}^{m} p_i\circ B(xy)}{2}. \end{aligned}$$

### **Theorem 26**

*Two isomorphic* *m*-*polar fuzzy graphs* $G_1=(V_1,A_1,B_1)$ *and* $G_2=(V_2,A_2,B_2)$ *of the graphs* $G^*_1=(V_1,E_1)$ *and* $G^*_2=(V_2,E_2)$ *have same order and size.*

### *Proof*

Let $\phi$ be an isomorphism from $G_1$ onto $G_2$. Then $A_1(x)=A_2(\phi (x))$ for all $x\in V_1$ and $p_i\circ B_1(xy)=p_i\circ B_2(\phi (x)\phi (y))$ for $i=1,2,\ldots ,m$, $xy\in \widetilde{V^2_1}$.

Now,

$$\begin{aligned} O(G_1)&=  {} |V_1|=\sum _{x\in V_1} \frac{1+ \sum \nolimits _{i=1}^{m} p_i\circ A_1(x)}{2} \\&=  {} \sum _{\phi (x)\in V_2} \frac{1+ \sum \nolimits _{i=1}^{m} p_i\circ A_2(\phi (x))}{2}=O(G_2) \end{aligned}$$

and

$$\begin{aligned} S(G_1)&=  {} |E_1|=\sum _{xy\in E_1} \frac{1+ \sum \nolimits _{i=1}^{m} p_i\circ B_1(xy)}{2} \\&=  {} \sum _{\phi(x)\phi(y)\in E_2} \frac{1+ \sum \nolimits _{i=1}^{m} p_i\circ B_2(\phi (x)\phi (y))}{2}=S(G_2). \end{aligned}$$

$$\square $$

### **Definition 27**

The busy value of a vertex *u* of an *m*-polar fuzzy graph *G* is denoted as $D(u)=(p_1\circ D(u),p_2\circ D(u),\ldots ,p_m\circ D(u))$ where $p_i\circ D(u)=\sum \limits _{k}min\{p_i\circ A(u),p_i\circ A(u_k)\}$; $u_k$ are the neighbors of *u*. The busy value of *G* is denoted as *D*(*G*) where $D(G)=\sum \limits _{k}D(u_k)$, $u_k\in V$.

### *Example 28*

Consider the 3-polar fuzzy graph $G=(V,A,B)$ of $G^*=(V,E)$ where $V=\{u,v,w,x\}$, $E=\{uv,vw,ux,uw,vx\}$, $A=\left\{ \frac{\langle 0.6,0.3,0.5\rangle }{u},\frac{\langle 0.8,0.4,0.3\rangle }{v},\, \frac{\langle 0.5,0.6,0.4\rangle }{w},\frac{\langle 0.7,0.5,0.6\rangle }{x}\right\}$ and $B=\left\{ \frac{\langle 0.5,0.2,0.2\rangle }{uv},\frac{\langle 0.1,0.3,0.2\rangle }{vw},\frac{\langle 0.6,0.2,0.4\rangle }{ux},\frac{\langle 0.3,0.2,0.3\rangle }{uw},\frac{\langle 0.7,0.4,0.2\rangle }{vx}\right\}$. Then we have from Fig. 5,

$$\begin{aligned}&p_1\circ D(u) = 1.7,\quad p_2\circ D(u)=0.9,\quad p_3\circ D(u)=1.2,\\&p_1\circ D(v) = 1.8,\quad p_2\circ D(v)=1.1,\quad p_3\circ D(v)=0.9,\\&p_1\circ D(w) = 1,\quad p_2\circ D(w)=0.7,\quad p_3\circ D(w)=0.7,\\&p_1\circ D(x) = 1.3,\quad p_2\circ D(x)=0.7,\quad p_3\circ D(x)=0.8. \end{aligned}$$

So, $$D(u)=(1.7,0.9,1.2), D(v) = (1.8,1.1,0.9), D(w)=(1,0.7,0.7), D(x)=(1.3,0.7,0.8)$$.

### **Definition 29**

If $p_i\circ A(u)\le p_i\circ deg(u)$ for $i=1,2,\ldots ,m$, then the vertex *u* of *G* is called a busy vertex. Otherwise it is a free vertex.

### **Definition 30**

If $p_i\circ B(u_1v_1)=min\{p_i\circ A(u_1),p_i\circ A(v_1)\}$, $i=1,2,\ldots ,m$ for $u_1v_1\in E$, then it is called an effective edge of *G*.

### **Definition 31**

Let $u\in V$ be a vertex of the *m*-polar fuzzy graph $G=(V,A,B)$.

(i) *u* is called a partial free vertex if it is a free vertex of *G* and $\overline{G}$.
(ii) *u* is called a fully free vertex if it is a free vertex of *G* and it is a busy vertex of $\overline{G}$.
(iii) *u* is called a partial busy vertex if it is a busy vertex of *G* and $\overline{G}$.
(iv) *u* is called a fully busy vertex if it is a busy vertex in *G* and it is a free vertex of $\overline{G}$.

### **Theorem 32**

*Let* $\phi$ *be an isomorphism from* $G_1=(V_1,A_1,B_1)$ *onto* $G_2=(V_2,A_2,B_2)$. *Then* $deg(u)=deg(\phi (u))$ *for all* $u\in V_1$.

### *Proof*

Since $\phi$ is an isomorphism between $G_1$ and $G_2$, we have $p_i\circ A_1(u)=p_i\circ A_2(\phi (u))$ for all $u\in V_1$ and $p_i\circ B_1(x_1y_1)=p_i\circ B_2(\phi (x_1)\phi (y_1))$ for all $x_1y_1\in \widetilde{V_1^2}$, $i=1,2,\ldots ,m$.

Hence, $$p_i\circ deg(u)=\sum \nolimits _{\begin{array}{c} u\ne v\\ uv\in E_1 \end{array}} p_i\circ B_1(uv) =\sum \nolimits _{\begin{array}{c} \phi (u)\ne \phi (v)\\ \phi (u)\phi (v)\in E_2 \end{array}} p_i\circ B_2(\phi (u)\phi (v)) =p_i\circ deg(\phi (u))$$ for $u\in V_1$, $i=1,2,\ldots ,m$. So, $deg(u)=deg(\phi (u))$ for all $u\in V_1$. $\square$

### **Theorem 33**

*If* $\phi$ *is an isomorphism from* $G_1$ *onto* $G_2$ *and* *u* *is a busy vertex of* $G_1$, *then* $\phi (u)$ *is a busy vertex of* $G_2$.

### *Proof*

Since $\phi$ is an isomorphism between we have, $p_i\circ A_1(u)=p_i\circ A_2(\phi (u))$ $u\in V_1$ and $p_i\circ B_1(x_1y_1)=p_i\circ B_2(\phi (x_1)\phi (y_1))$ for $x_1y_1\in \widetilde{V_1^2}$, $i=1,2,\ldots ,m$.

If *u* is a busy vertex of $G_1$, then $p_i\circ A_1(u)\le p_i\circ deg(u)$ for $i=1,2,\ldots ,m$. Then by the above and Theorem 32, $p_i\circ A_2(\phi (u))=p_i\circ A_1(u)\le p_i\circ deg(u)=p_i\circ deg(\phi (u))$ for $i=1,2,\ldots ,m$. Hence, $\phi (u)$ is a busy vertex in $G_2$. $\square$

### **Theorem 34**

*Let the two* *m*-*polar fuzzy graphs* $G_1$ *and* $G_2$ *be weak isomorphic. If* $u\in V_1$ *is a busy vertex of* $G_1$, *then the image of* *u* *under the weak isomorphism is also busy in* $G_2$.

### *Proof*

Let $\phi :V_1\rightarrow V_2$ be a weak isomorphism between $G_1$ and $G_2$.

Then, $p_i\circ A_1(x)=p_i\circ A_2(\phi (x))$ for all $x\in V_1$ and $p_i\circ B_1(x_1y_1)\le p_i\circ B_2(\phi (x_1)\phi (y_1))$ for all $x_1y_1\in \widetilde{V_1^2}$, $i=1,2,\ldots ,m$.

Let $u\in V_1$ be a busy vertex. Then, for $i=1,2,\ldots ,m$, $p_i\circ A_1(u)\le p_i\circ deg(u)$.

Now by the above for $i=1,2,\ldots ,m$

$$\begin{aligned} p_i\circ A_2(u)&=  {} p_i\circ A_1(u)\le p_i\circ deg(u)=\sum \limits _{\begin{array}{c} u\ne v\\ uv\in E_1 \end{array}} p_i\circ B_1(uv)\\&\le  {} \sum \limits _{\begin{array}{c} \phi (u)\ne \phi (v)\\ \phi (u)\phi (v)\in E_2 \end{array}} p_i\circ B_2(\phi (u)\phi (v)) =p_i\circ deg(\phi (u)). \end{aligned}$$

Hence, $\phi (u)$ is a busy vertex in $G_2$. $\square$

## Complement and isomorphism in *m*-polar fuzzy graphs

In this section some important properties of isomorphism, weak isomorphism, co weak isomorphism related with complement are discussed.

### **Theorem 35**

*Let* $G_1=(V_1,A_1,B_1)$ *and* $G_2=(V_2,A_2,B_2)$ *be two* *m*-*polar fuzzy graphs of the graphs* $G^*_1=(V_1,E_1)$ *and* $G^*_2=(V_2,E_2)$. *If* $G_1\cong G_2$ *then* $\overline{G_1}\cong \overline{G_2}$.

### *Proof*

Let $G_1\cong G_2$. Then there exists an isomorphism $\phi : V_1\rightarrow V_2$ such that $A_1(x)=A_2(\phi (x))$ for all $x\in V_1$ and $p_i\circ B_1(xy)=p_i\circ B_2(\phi (x)\phi (y))$, for each $i=1,2,\ldots ,m$ and $xy\in \widetilde{V^2_1}$.

Now, $\overline{A_1}(x)=A_1(x)=A_2(\phi (x))=\overline{A_2}(\phi (x))$ for all $x\in V_1$.

Also, for $i=1,2,\ldots ,m$ and $xy\in \widetilde{V^2_1}$ we have,

$$\begin{aligned} p_i\circ \overline{B_1}(xy)&=  {} min\{p_i\circ A_1(x),p_i\circ A_1(y)\}-p_i\circ B_1(xy)\\&=  {} min\{p_i\circ A_2(\phi (x),p_i\circ A_2(\phi (y)\}-p_i\circ B_2(\phi (x)\phi (y))=p_i\circ \overline{B_2}(\phi (x)\phi (y)). \end{aligned}$$

Hence, $\phi$ is an isomorphism between $\overline{G_1}$ and $\overline{G_2}$ i.e., $\overline{G_1}\cong \overline{G_2}$. $\square$

### *Remark 36*

Suppose there is a weak isomorphism between two *m*-polar fuzzy graphs $G_1$ and $G_2$. Then there may not be a weak isomorphism between $\overline{G_1}$ and $\overline{G_2}$.

For example, consider two 3-polar fuzzy graphs $G_1$ and $G_2$ of Fig. 6. Let us now define a mapping $\phi : V_1 \rightarrow V_2$ such that $\phi (a)=u$, $\phi (b)=v$, $\phi (c)=w$. Then $\phi$ is a weak isomorphism from $G_1$ onto $G_2$. But, there is no weak isomorphism from $\overline{G_1}$ onto $\overline{G_2}$ (see Fig. 7) because $\overline{B_2}(uw=\phi (a)\phi (c))={\mathbf{0}} =(0,0,\ldots ,0)<\overline{B_1}(ac)=(0.1,0.1,0.05)$, and $\overline{B_2}(vw=\phi (b)\phi (c))={\mathbf{0}} =(0,0,\ldots ,0)<\overline{B_1}(bc)=(0.1,0.1,0.1)$.

### *Remark 37*

In a similar way, we can construct example to show that if there is a co-weak isomorphism between two *m*-polar fuzzy graphs $G_1$ and $G_2$ then there may not be a co-weak isomorphism between $\overline{G_1}$ and $\overline{G_2}$.

### **Theorem 38**

*Let* $G_1=(V_1,A_1,B_1)$ *and* $G_2=(V_2,A_2,B_2)$ *be two* *m*-*polar fuzzy graphs of the graphs* $G^*_1=(V_1,E_1)$ *and* $G^*_2=(V_2,E_2)$ *such that* $V_1\cap V_2= \emptyset$. *Then* $\overline{G_1+G_2}\cong \overline{G_1}\cup \overline{G_2}$.

### *Proof*

To show that $\overline{G_1+G_2}\cong \overline{G_1}\cup \overline{G_2}$, we need to show that there exists an isomorphism between $\overline{G_1+G_2}$ and $\overline{G_1}\cup \overline{G_2}$.

We will show that the identity map $I:V_1\cup V_2 \rightarrow V_1\cup V_2$ is the required isomorphism between them. For this, we will show the following:

for all $$x\in V_1\cup V_2$$, $$\overline{(A_1+A_2)}(x)=(\overline{A_1}\cup \overline{A_2})(x)$$,

and $p_i\circ \overline{(B_1+B_2)}(xy)=p_i\circ (\overline{B_1}\cup \overline{B_2})(xy)$ for $i=1,2,\ldots ,m$ and $xy\in \widetilde{V_1\times V_2}^2$.

Let $$x\in V_1\cup V_2$$.

Then

$$\begin{aligned} \overline{(A_1+A_2)}(x)&=  {} (A_1+A_2)(x)=(A_1\cup A_2)(x)\quad ({\text {by Definition }}8)\\&=  {} \left\{ \begin{array}{ll} A_1(x) &{}\quad {\text {if}}\; x\in V_1-V_2\\ A_2(x) &{}\quad {\text {if}}\; x\in V_2-V_1\\ \end{array}\right. \\&=  {} \left\{ \begin{array}{ll} \overline{A_1}(x) &{}\quad {\text {if}}\; x\in V_1-V_2\\ \overline{A_2}(x) &{}\quad {\text {if}}\; x\in V_2-V_1\\ \end{array}\right. =(\overline{A_1}\cup \overline{A_2})(x). \end{aligned}$$

Now for each $i=1,2,\ldots ,m$ and $xy\in \widetilde{V_1\times V_2}^2$ we have,

$$\begin{aligned}&p_i\circ \overline{(B_1+B_2)}(xy)\\&\quad =min\{p_i\circ (A_1+A_2)(x),p_i\circ (A_1+A_2)(y)\}-p_i\circ (B_1+B_2)(xy)\\&\quad = \left\{ \begin{array}{ll} min\{p_i\circ (A_1\cup A_2)(x),p_i\circ (A_1\cup A_2)(y)\}-p_i\circ (B_1\cup B_2)(xy), &{}\quad {\text {if}}\; xy\in E_1\cup E_2\\ min\{p_i\circ (A_1\cup A_2)(x),p_i\circ (A_1\cup A_2)(y)\}-min\{p_i\circ A_1(x),p_i\circ A_2(y)\}, &{}\quad {\text {if}}\; xy\in E^\prime \\ \end{array}\right. \\&\quad = \left\{ \begin{array}{ll} min\{p_i\circ A_1(x),p_i\circ A_1(y)\}-p_i\circ B_1(xy), &{}\quad {\text {if}}\; xy\in E_1-E_2\\ min\{p_i\circ A_2(x),p_i\circ A_2(y)\}-p_i\circ B_2(xy), &{}\quad {\text {if}}\; xy\in E_2-E_1\\ min\{p_i\circ A_1(x),p_i\circ A_2(y)\}-min\{p_i\circ (A_1)(x),p_i\circ (A_2)(y)\}, &{}\quad {\text {if}}\; xy\in E^\prime \\ \end{array}\right. \\&\quad = \left\{ \begin{array}{ll} p_i\circ \overline{B_1}(xy), &{}\quad {\text {if}}\; xy\in E_1-E_2\\ p_i\circ \overline{B_2}(xy), &{}\quad {\text {if}}\; xy\in E_2-E_1\\ 0, &{}\quad {\text {if}}\; xy\in E^\prime \\ \end{array}\right. \\&\quad = p_i\circ (\overline{B_1}\cup \overline{B_2})(xy). \end{aligned}$$

$$\square $$

### **Theorem 39**

*Let* $G_1=(V_1,A_1,B_1)$ *and* $G_2=(V_2,A_2,B_2)$ *be two* *m*-*polar fuzzy graphs of the graphs* $G^*_1=(V_1,E_1)$ *and* $G^*_2=(V_2,E_2)$ *such that* $V_1\cap V_2= \emptyset$. *Then* $\overline{G_1\cup G_2}\cong \overline{G_1}+\overline{G_2}$.

### *Proof*

Consider the identity map $I:V_1\cup V_2\rightarrow V_1\cup V_2$. We will show that *I* is the required isomorphism between $\overline{G_1\cup G_2}$ and $\overline{G_1}+\overline{G_2}$.

For this, we will show the following:

for all $$x\in V_1\cup V_2$$, $$\overline{(A_1\cup A_2)}(x)=(\overline{A_1}+\overline{A_2})(x)$$,

and $p_i\circ \overline{(B_1\cup B_2)}(xy)=p_i\circ (\overline{B_1}+\overline{B_2})(xy)$ for $i=1,2,\ldots ,m$ and $xy\in \widetilde{V_1\times V_2}^2$.

Let $$x\in V_1\cup V_2$$.

Then

$$\begin{aligned} \overline{A_1\cup A_2}(x)&=  {} (A_1\cup A_2)(x)\\&=  {} \left\{ \begin{array}{ll} A_1(x), &{}\quad {\text {if}}\; x\in V_1-V_2\\ A_2(x), &{}\quad {\text {if}}\; x\in V_2-V_1\\ \end{array}\right. \\&=  {} \left\{ \begin{array}{ll} \overline{A_1}(x), &{}\quad {\text {if}}\; x\in V_1-V_2\\ \overline{A_2}(x), &{}\quad {\text {if}}\; x\in V_2-V_1\\ \end{array}\right. \\&=  {} (\overline{A_1}\cup \overline{A_2})(x) \end{aligned}$$

and for $i=1,2,\ldots ,m$, $xy\in \widetilde{V_1\times V_2}^2$ we have,

$$\begin{aligned}&p_i\circ \overline{(B_1\cup B_2)}(xy)\\&\quad =min\{p_i\circ (A_1\cup A_2)(x),p_i\circ (A_1\cup A_2)(y)\}-p_i\circ (B_1\cup B_2)(xy)\\&\quad =\left\{ \begin{array}{ll} min\{p_i\circ A_1(x),p_i\circ A_1(y)\}-p_i\circ B_1(xy), &{}\quad {\text {if}}\; xy\in E_1- E_2\\ min\{p_i\circ A_2(x),p_i\circ A_2(y)\}-p_i\circ B_2(xy), &{}\quad {\text {if}}\; xy\in E_2-E_1\\ min\{p_i\circ A_1(x),p_i\circ A_2(y)\}-0, &{}\quad {\text {if}}\; x\in V_1,y\in V_2\\ \end{array}\right. \\&\quad =\left\{ \begin{array}{ll} p_i\circ \overline{B_1}(xy), &{}\quad {\text {if}}\; xy\in E_1-E_2\\ p_i\circ \overline{B_2}(xy), &{}\quad {\text {if}}\; xy\in E_2-E_1\\ min\{p_i\circ A_1(x),p_i\circ A_2(y)\}-0, &{}\quad {\text {if}}\; x\in V_1,y\in V_2\\ \end{array}\right. \\&\quad =\left\{ \begin{array}{ll} p_i\circ \overline{B_1}(xy), &{}\quad {\text {if}}\; xy\in E_1-E_2\\ p_i\circ \overline{B_2}(xy), &{}\quad {\text {if}}\; xy\in E_2-E_1\\ min\{p_i\circ A_1(x),p_i\circ A_2(y)\}-0, &{}\quad {\text {if}}\; xy\in E^\prime \\ \end{array}\right. \\&\quad =p_i\circ (\overline{B_1}+\overline{B_2})(xy). \end{aligned}$$

This completes the proof. $\square$

### **Theorem 40**

*Let* $G_1=(V_1,A_1,B_1)$ *and* $G_2=(V_2,A_2,B_2)$ *be two strong* *m*-*polar fuzzy graphs of the graphs* $G^*_1=(V_1,E_1)$ *and* $G^*_2=(V_2,E_2)$ *respectively*. *Then* $\overline{G_1\circ G_2}\cong \overline{G_1}\circ \overline{G_2}$.

### *Proof*

Let $G_1\circ G_2=(V_1\times V_2,A_1\circ A_2,B_1\circ B_2)$ be an *m*-polar fuzzy graph of the graph $G^*=(V,E)$ where $V=V_1\times V_2$ and $E=\{(x,x_2)(x,y_2): x\in V_1, x_2y_2\in E_2\}\cup \{(x_1,z)(y_1,z): z\in V_2, x_1y_1\in E_1\}\cup \{(x_1,x_2)(y_1,y_2): x_1y_1\in E_1, x_2\ne y_2\}$.

We show that the identity map *I* is the required isomorphism between the graphs $\overline{G_1\circ G_2}$ and $\overline{G_1}\circ \overline{G_2}$. Let us consider the identity map $I: V_1\times V_2 \rightarrow V_1\times V_2$.

In order to show that *I* is the required isomorphism, we show that for each $i=1,2,\ldots ,m$ and for all $xy\in \widetilde{V_1\times V_2}^2$, $p_i\circ \overline{(B_1\circ B_2)}(xy)=p_i\circ (\overline{B_1} \circ \overline{B_2})(xy)$. Several cases may arise.

- *Case* (*i*): Let $e=(x,x_2)(x,y_2)$ where $x\in V_1$, $x_2y_2\in E_2$. Then $e\in E$.
  Since $G_1\circ G_2$ is strong *m*-polar fuzzy graph, we have for each $i=1,2,\ldots ,m$
  $$\begin{aligned}&p_i\circ \overline{(B_1\circ B_2)}(e)=0{\text { and}}\\&p_i\circ (\overline{B_1} \circ \overline{B_2})(e)=min\{p_i\circ A_1(x),p_i\circ \overline{B_2}(x_2y_2)\}=0 \end{aligned}$$
  (since $G_2$ is strong and $x_2y_2\in E_2$, therefore for each $i=1,2,\ldots ,m$, $p_i\circ \overline{B_2}(x_2y_2)=0$).
- *Case* (*ii*): Let $e=(x,x_2)(x,y_2)$ where $x_2\ne y_2$, $x_2y_2\notin E_2$. Then $e\notin E$.
  So for each $i=1,2,\ldots ,m$, $p_i\circ (B_1\circ B_2)(e)=0$ and
  $$\begin{aligned} p_i\circ \overline{(B_1\circ B_2)}(e)&=  {} min\{p_i\circ (A_1\circ A_2)(x,x_2),p_i\circ (A_1\circ A_2)(x,y_2)\}\\&=  {} min\{p_i\circ A_1(x),p_i\circ A_2(x_2),p_i\circ A_2(y_2)\}. \end{aligned}$$
  Again, since $x_2y_2\in \overline{E_2}$, therefore for each $i=1,2,\ldots ,m$,
  $$\begin{aligned} p_i\circ (\overline{B_1} \circ \overline{B_2})(e)&=  {} min\{p_i\circ A_1(x),p_i\circ \overline{B_2}(x_2y_2)\}\\&=  {} min\{p_i\circ A_1(x),p_i\circ A_2(x_2),p_i\circ A_2(y_2)\}. \end{aligned}$$
- *Case* (*iii*): Let $e=(x_1,z)(y_1,z)$ where $x_1y_1\in E_1$, $z\in V_2$.
  Then $e\in E$. So for each $i=1,2,\ldots ,m$, $p_i\circ \overline{(B_1\circ B_2)}(e)=0$ as in Case (i).
  Also, since $x_1y_1\notin \overline{E_1}$, therefore for each $i=1,2,\ldots ,m$, $p_i\circ (\overline{B_1}\circ \overline{B_2})(e)=0$.
- *Case* (*iv*): Let $e=(x_1,z)(y_1,z)$ where $x_1y_1\notin E_1$, $z\in V_2$. Then $e\notin E$.
  Hence for each $i=1,2,\ldots ,m$, $p_i\circ (B_1\circ B_2)(e)=0$,
  $$\begin{aligned} p_i\circ \overline{(B_1\circ B_2)}(e)&=  {} min\{p_i\circ (A_1\circ A_2)(x_1,z),p_i\circ (A_1\circ A_2)(y_1,z)\}\\&=  {} min\{p_i\circ A_1(x_1),p_i\circ A_1(y_1),p_i\circ A_2(z)\}\text { and}\\ p_i\circ (\overline{B_1} \circ \overline{B_2})(e)&=  {} min\{p_i\circ A_2(z),p_i\circ \overline{B_1}(x_1y_1)\}\\&=  {} min\{p_i\circ A_1(x_1),p_i\circ A_1(y_1),p_i\circ A_2(z)\} \;(G_1{\text { being strong}}). \end{aligned}$$
- *Case* (*v*): Let $e=(x_1,x_2)(y_1,y_2)$ where $x_1y_1\in E_1$, $x_2\ne y_2$. Then $e\in E$. So we have for each $i=1,2,\ldots ,m$, $p_i\circ \overline{(B_1\circ B_2)}(e)=0$ as in Case (i).
  Also, since $x_1y_1\in E_1$, we have for each $i=1,2,\ldots ,m$, $p_i\circ (\overline{B_1} \circ \overline{B_2})(e)=0$.
- *Case* (*vi*): Let $e=(x_1,x_2)(y_1,y_2)$ where $x_1y_1\notin E_1$, $x_2\ne y_2$. Then $e\notin E$ and hence for each $i=1,2,\ldots ,m$, $p_i\circ (B_1\circ B_2)(e)=0$,
  $$\begin{aligned} p_i\circ \overline{(B_1\circ B_2)}(e)&=  {} min\{p_i\circ (A_1\circ A_2)(x_1,x_2),p_i\circ (A_1\circ A_2)(y_1,y_2)\}\\&=  {} min\{p_i\circ A_1(x_1),p_i\circ A_1(y_1),p_i\circ A_2(x_2),p_i\circ A_2(y_2)\} \end{aligned}$$
  and since $$x_1y_1\in \overline{E_1}$$,
  $$\begin{aligned} p_i\circ (\overline{B_1} \circ \overline{B_2})(e)&=  {} min\{p_i\circ A_2(x_2),p_i\circ A_2(y_2),p_i\circ \overline{B_1}(x_1y_1)\}\\&=  {} min\{p_i\circ A_1(x_1),p_i\circ A_1(y_1),p_i\circ A_2(x_2),p_i\circ A_2(y_2)\}\, (\overline{G_1} \text { being strong by } [10]). \end{aligned}$$
- *Case* (*vii*): Finally, let $e=(x_1,x_2)(y_1,y_2)$ where $x_1y_1\notin E_1$, $x_2y_2\notin E_2$. Then $e\notin E$ and hence for each $i=1,2,\ldots ,m$, $p_i\circ (B_1\circ B_2)(e)=0$,
  $$\begin{aligned} p_i\circ \overline{(B_1\circ B_2)}(e)=min\{p_i\circ (A_1\circ A_2)(x_1,x_2),p_i\circ (A_1\circ A_2)(y_1,y_2)\}. \end{aligned}$$
  Now, $x_1y_1\in \overline{E_1}$ and if $x_2=y_2=z$, then we have the Case (iv).
  Again, if $x_1y_1\in \overline{E_1}$ and if $x_2\ne y_2$, then we have Case (vi).
  Thus combining all the cases we have, for each $i=1,2,\ldots ,m$, and $xy\in \widetilde{V_1\times V_2}^2$,
  $$\begin{aligned} p_i\circ \overline{(B_1\circ B_2)}(xy)=p_i\circ (\overline{B_1} \circ \overline{B_2})(xy). \end{aligned}$$
  $$\square $$

### *Remark 41*

If $G_1$ and $G_2$ are not strong, then $\overline{G_1\circ G_2} \ncong \overline{G_1}\circ \overline{G_2}$ always. For example, consider the two 3-polar fuzzy graphs $G_1$ and $G_2$ which are not strong (see Fig. 8). From Figs. 8 and 9, we see that, $\overline{G_1\circ G_2}\ncong \overline{G_1}\circ \overline{G_2}$.

## Applications

Now a days, fuzzy graphs and bipolar fuzzy graphs are most familiar graphs to us and they can also be thought of as 1-polar and 2-polar fuzzy graphs respectively. These graphs have many important application in social networks, medical diagnosis, computer networks, database theory, expert system, neural networks, artificial intelligence, signal processing, pattern recognition, engineering science, cluster analysis, etc. The concepts of bipolar fuzzy graphs can be generalized to *m*-polar fuzzy graphs. For example, consider the sorting of mangoes and guavas. Now the different characteristics of a given fruit can change the decision in sorting process more towards the decision mango or vice versa. There are two poles present in this case. One is $100\%$ sure mango and the other is $100\%$ sure guava. This shows that the situation is bipolar. This situation can be generalized further by adding a new fruit, for example sweet lemon into the sorting process.

### Graphical representation of tug of war

Consider the another example of tug of war where two people pull the rope in opposite directions. Here, who uses the bigger force, the center of the rope will move in the respective direction of their pulling. The situation is symmetric in this case. We present an example where *m* people pull a special rope in *m* different directions. We use this example to represent it as an *m*-polar fuzzy graph. We assume that *O* is the origin and there are *m* straight paths leading from *O*. We also assume that there is a wall in between these paths. In this setting, we have the special rope with one node at *O* and *m* endings going out from this nodes—one end corresponding to each of the paths. Suppose on every path there is a man standing and pulling the rope in the direction of the path on which he is standing. This situation can be represented as an *m*-polar fuzzy graph by considering the nodes as *m*-polar fuzzy set and edges between them as *m*-polar fuzzy relations, which is shown in Fig. 10. In this context, one can ask the question what is the strength require in order to pull the node *O* from the center into one of the paths (assuming no friction)? The answer to this is that if the corresponding forces which are pulling the rope are $F_k$, $k=1,2,\ldots ,m$, then the node *O* will move to the $j\hbox {th}$ path if $$F_j > \sum \nolimits _{\begin{array}{c} k=1,2,\ldots ,m\\ k\ne j \end{array}}{F_k}$$.

### Evaluation graph corresponding to the teacher’s evaluation by the students

In this section we present the model of *m*-polar fuzzy graph which is used in evaluating the teachers by the students of 4th semester of a department in an university during the session 2015–2016. Here the nodes represent the teachers of the corresponding department and edges represent the relationship between two teachers. Suppose the department has six teachers denoted as $T=\{t_1,t_2,t_3,t_4,t_5,t_6\}$. The membership value of each node represents the corresponding teachers feedback response of the students depending on the following: {*regularity of classes, style of presentation, quality of lectures, generation of interest and encouraging future reading among students, updated information*}. Since all the above characteristics of a teacher according to the different students are uncertain in real life, therefore we consider 5-polar fuzzy subset of the vertex set *T* (Fig. 11).

In the Table 1, the membership values of the teacher’s are given which is according to the evaluation of the students.

**Table 1:** 5-Polar fuzzy set *A* of *T*

	$t_1$	$t_2$	$t_3$	$t_4$	$t_5$	$t_6$
$p_1\circ A$	0.6	0.7	0.8	0.8	0.8	0.7
$p_2\circ A$	0.7	0.6	0.9	0.7	0.9	0.8
$p_3\circ A$	0.8	0.7	0.7	0.8	0.7	0.9
$p_4\circ A$	0.9	0.8	0.8	0.9	0.7	0.7
$p_5\circ A$	0.9	0.8	0.9	0.8	0.8	0.8

**Table 2:** 5-Polar fuzzy relation *B* on *A*

	$t_1t_2$	$t_1t_5$	$t_1t_6$	$t_2t_3$	$t_2t_4$	$t_2t_5$	$t_3t_4$	$t_3t_5$	$t_4t_5$	$t_4t_6$	$t_5t_6$
$p_1\circ A$	0.6	0.5	0.6	0.6	0.8	0.7	0.8	0.7	0.8	0.6	0.7
$p_2\circ A$	0.6	0.7	0.7	0.6	0.7	0.5	0.7	0.5	0.7	0.7	0.8
$p_3\circ A$	0.7	0.7	0.8	0.6	0.6	0.6	0.7	0.7	0.7	0.7	0.6
$p_4\circ A$	0.8	0.6	0.7	0.7	0.6	0.7	0.8	0.7	0.7	0.7	0.6
$p_5\circ A$	0.7	0.8	0.8	0.8	0.8	0.6	0.8	0.8	0.8	0.7	0.7

**Table 3:** Average response score of the teachers

	Teachers					
	$t_1$	$t_2$	$t_3$	$t_4$	$t_5$	$t_6$
*Scores*	0.78	0.72	0.82	0.8	0.78	0.78

Edge membership values which represent the relationship between the teachers can be calculated by using the relation $p_i\circ B(uv)\le min\{p_i\circ A(u), p_i\circ A(v)\}$ for all $u,v\in T$, $i=1,2,\ldots ,5$. These values are given in the Table 2.

We rank the teacher’s performance according the following:

- Teacher’s average response score <60%, teacher’s performance according to the students is $\mathbf{Average}$.
- Teacher’s average response score ≥60% and <70%, teacher’s performance according to the students is $\mathbf{Good}$.
- Teacher’s average response score ≥70% and <80%, teacher’s performance according to the students is $\mathbf {Very}$ $\mathbf{Good}$.
- Teacher’s average response score is ≥80%, teacher’s performance according to the students is $\mathbf{Excellent}$.

From the Table 3, we see that the performance of the teachers $t_1,t_2,t_5,t_6$ are very good whereas the performance of the teachers $t_3$ and $t_4$ are excellent. Among these teachers, teacher $t_3$ is the best teacher according the response score of the students of the department during the session 2015–2016.

## Conclusions

The theory of fuzzy graphs play an important role in many fields including decision makings, computer networking and management sciences. An m-polar fuzzy graph can be used to represent real world problems which involve multi-agent, multi-attribute, multi-object, multi-index, multi-polar information and uncertainty. In this research paper, we have studied the isomorphic properties of *m*-polar fuzzy graphs with some applications. We are extending our research work on *m*-polar fuzzy intersection graphs, *m*-polar fuzzy interval graphs, properties of *m*-polar fuzzy hypergraphs, degrees of vertices of *m*-polar fuzzy graphs and its application in decision making, etc.
